# The Hon. Charles Benjamin Mosse

**Published:** 1912-07-13

**Authors:** 


### Obituary.

The Hon. Charles Benjamin Mosse, C.B., C.M.G., late Deputy Surgeon-General A.M.D., has died at Guernsey, aged eighty-two. For nearly twenty-five years he was Superintending Medical Officer, Colonial Medical Department, Jamaica. He served in the expedition up the River Gambia, West Africa, in June 1866. In 1867 he was promoted to staff-surgeon for "valuable services" during an epidemic of yellow fever at Bathurst. He served throughout the Ashanti War, 1873-4, and was made a C.B. It was then that he became Superintending Medical Officer for Jamaica in June 1896, and from which post he retired in 1904. He was a member of the Royal College of Surgeons, England, and of the Royal College of Physicians, Ireland. He qualified in 1852.

